# Lysophosphatidic Acid Analogue rather than Lysophosphatidic Acid Promoted the Bone Formation In Vivo

**DOI:** 10.1155/2018/7537630

**Published:** 2018-05-29

**Authors:** Zi-Li Yu, Bin-Fang Jiao, Zu-Bing Li

**Affiliations:** ^1^The State Key Laboratory Breeding Base of Basic Science of Stomatology (Hubei-MOST) and Key Laboratory of Oral Biomedicine Ministry of Education, School and Hospital of Stomatology, Wuhan University, 237 Luoyu Road, Wuhan, China; ^2^Department of Oral and Maxillofacial Surgery, School and Hospital of Stomatology, Wuhan University, 237 Luoyu Road, Wuhan, China; ^3^Wuhan No. 1 Stomatological Hospital, 675 Jianshe Avenue, Wuhan, China

## Abstract

Lysophosphatidic acid (LPA), a bioactive lipid molecule, has recently emerged as physiological and pathophysiological regulator in skeletal biology. Here we evaluate the effects of LPA on bone formation in vivo in murine femoral critical defect model. Primary femoral osteoblasts were isolated and treated with osteogenic induction conditional media supplemented with 20 *μ*M LPA or LPA analogue. Mineralized nodules were visualized by Alizarin Red S staining. Forty-five C57BL/6 mice underwent unilateral osteotomy. The femoral osteotomy gap was filled with porous scaffolds of degradable chitosan/beta-tricalcium phosphate containing PBS, LPA, or LPA analogue. 2, 5, and 10 weeks after surgery, mice were sacrificed and femurs were harvested and prepared for Micro-Computed Tomography (Micro-CT) and histological analysis. Alizarin Red S staining showed that LPA and LPA analogue significantly enhanced the mineral deposition in osteoblasts. Micro-CT 3D reconstruction images and HE staining revealed that significantly more newly formed bone in osteotomy was treated with LPA analogue when compared to control and LPA group, which was verified by histological analysis and biomechanical characterization testing. In summary, our study demonstrated that although LPA promotes mineralized matrix formation in vitro, the locally administrated LPA was not effective in promoting bone formation in vivo. And bone formation was enhanced by LPA analogue, administrated locally in vivo. LPA analogue was a potent stimulating factor for bone formation in vivo due to its excellent stability.

## 1. Introduction

Fractures are the most common traumatic tissue injury in humans and nearly 10% fractures will not heal normally [1]. Osteoinductive molecules can act as external bone formation stimulators and offer a promising approach for nonunion therapy. Among them, bone morphogenic proteins 2 (BMP-2) have been approved for clinical application by Food and Drug Administration (FDA) because of their successfully osteoinductive effects and safety. However, high BMP protein prices may act as a drag on household budgets, pushing more people in developing country into poverty. Moreover, the complicated preparation process, long production cycle, low yield, and high price prevent its large scale production [2]. Therefore, it is highly desirable to develop a reliable BMP2 substitute with abundant resources, low price, and excellent osteoinductive effects. Lysophosphatidic acid (LPA) is the simplest signaling lipid molecule that has pleiotropic effects upon most mammalian cell types. According to previous studies, LPA has an established role in a series of biological processes, especially wound healing and cancer [3–8]. It was reported that LPA is found in mammalian serum and plasma, present at the levels of 1-20 *μ*M. Most importantly, the concentration of LPA was elevated significantly at sites of tissue injury [9, 10]. For example, nerve injury caused high levels of LPA production in the ipsilateral sides of the spinal dorsal horn and dorsal roots [11]. Those findings suggested that LPA may play a role in tissue injury. LPA originate from activated platelets and other blood-borne cells, including mast cells participates in the nature wound-healing process by stimulating cell proliferation at the site of injury [9]. Recently, the emerging role of LPA in skeletal biology has gained considerable scientific interest [12].

It is well accepted that the bone healing process is mediated by numerous biomolecules. A hematoma is formed in the injured site when fractures happened [13, 14]. On the other hand, aggregation and activation of platelets result in the rapid release of LPA into the extracellular environment [9, 12]. LPA receptors are expressed on osteoblasts and LPA elicits its bioactive functions through receptors on osteoblasts cytomembrane [4, 15–17]. Recently, researchers found that the activation of P2X7 receptors expressed in osteoblasts will enhance the production of LPA [18]. Moreover, LPA promote osteoblasts proliferation [19, 20] and inhibit cell apoptosis [21]. Most importantly, LPA is capable of inducing osteogenesis of human mesenchymal stem cell (hMSC) [5] and increasing the expression of osteoblast marker genes and the mineralization level of osteoblasts [18]. LPA receptor-deficient mice have increased trabecular bone volume, number, and thickness [5].

These exciting observations mentioned above suggested that LPA may play an important role in bone healing and targeting LPA or LPA receptors may provide new avenues to enhance the bone fracture healing. Regrettably, up to now, to our knowledge, there are no studies dealing with the effect of exogenous LPA on bone formation in vivo. This could be due to the labile nature of LPA [22]. In this study, we synthesized the LPA analogue which was more stable than LPA and evaluated the effect of LPA and LPA analogue on bone formation in murine femoral critical defect model. We found that LPA analogue rather than LPA significantly accelerated the bone healing process in vivo.

## 2. Materials and Methods

### 2.1. Regents

All consumables used for cell culture were purchased from Corning (Acton, MA). Cell culture media, fetal bovine serum (FBS), and buffers were purchased from Thermo Scientific HyClone (Waltham, MA, USA). Mouse anti-BMP2, runt-related transcription factor 2 (RUNX2), ALP, osteocalcin (OCN), and *β*-Actin antibody were obtained from Santa Cruz. Secondary antibodies were purchased from Beijing Hapten and Protein Biomedical Institute (Beijing, China). 1-Oleoyl lysophosphatidic acid (sodium salt) was purchased from Cayman Chemical (Iten No. 62215; Michigan, USA). LPA analogue was synthesized as described previously [23, 24]. The monofluorinated and difluorinated LPA analogues in which either the sn-1 or sn-2 hydroxy group was replaced by fluorine were prepared from (R)- or (S)-solketal. The fluorine substitutes blocks acyl migration, effectively “freezing” them in the sn-1-O-acyl or sn-2-O-acyl forms. The fluoromethylene phosphonate LPA analogues were prepared from 3,4-epoxy butylfluorophosphonate using a chiral salen-Co catalyzed HKR (hydrolytic kinetic resolution) reaction.

### 2.2. Preparation of Porous Scaffolds of Degradable Chitosan/B-Tricalcium Phosphate

The porous scaffolds of degradable chitosan/beta-tricalcium phosphate (CS/*β*-TCP) in a ratio of 4:2.7 were prepared according to our previously reported methods [25]. Chitosan solutions with concentrations of 2 wt % were prepared by the dissolution of chitosan in a 0.2 M acetic acid. The mixture was stirred at 50°C for 2 h to obtain a homogeneous polymer solution and was filtered through a fine cloth to remove the air bubbles trapped in the viscous liquid. The *β*-TCP powders were then added to the prepared solution to make a polymer/calcium phosphate mixture. The final composition of the composite foam was determined by the concentration of the chitosan solution and calcium phosphate content in the mixture. The mixture was then rapidly transferred into a freezer at a preset temperature of −20°C to solidify the solvent and induce a solid-liquid phase separation. The solidified mixture was maintained at −20°C for 8 h, and the frozen mixture was then transferred into a freeze dryer at a preset temperature of −5°C. The samples were freeze-dried at 0.5 mmHg for at least 4 days to completely remove the solvent.

### 2.3. Isolation and Culture of Primary Osteoblasts

All animal procedures were approved by the Animal Care and Use Committee of the Hospital of Stomatology, Wuhan University (Approval number: 2012-33). Femur osteoblasts were isolated from five C57BL/6 mice (6 weeks old) by using enzymatic digestion as described previously [26]. Briefly, when epiphyses were cut off, thoroughly flush out bone marrow with sterile PBS. Then, cut the diaphyses into little pieces of approximately 1-2 mm^2^ using scissors. The little pieces were digested with collagenase II solution in shaking water bath at 37°C for 2 h. After washing with *α*-MEM medium for three times, the bone pieces were transferred to culture flask with 5 ml *α*-MEM medium containing 10% FBS and 100 *μ*g/mL penicillin/streptomycin. The culture medium was replaced three times per week.

### 2.4. Cell Viability Measurement

The cell viability of osteoblasts was performed as previously described [27]. Briefly, after incubation with LPA (20 *μ*M) or LPA analogue (20 *μ*M) for indicated time (0-72 h), the cell viability of osteoblasts was measured with cell viability analyzer (Beckman Coulter, Fullerton).

### 2.5. Wound-Healing Assay

Osteoblasts were seeded in the 6-well plate at 1 × 10^6^/well. When cells were grown to 80%-90% confluence, the monolayer of cells was scraped with a sterile 200-*μ*L micropipette tip. Then, the cells were washed with PBS and cultured in 2% serum media with BMP2 (30 ng/mL), LPA, or LPA analogue at various concentrations for 12 h. The migrated cells were counted under a phase microscope (Leica).

### 2.6. Transwell Assay

Transwell analysis was performed with Transwell Boyden chamber system (Corning Life Sciences) containing a polycarbonate filter (6.5 mm diameter; pore size of 8 *μ*m) according to previous study [28]. Osteoblasts were seeded on the upper chamber and treated with BMP2 (30 ng/mL), LPA, or LPA analogue with various concentrations at 37°C for 12 h. Culture medium with 10% FBS was applied to the lower chamber as chemoattractant. Then the cells on the surface of upper chamber were removed by a cotton swab. The migrated cells were fixed with 4% paraformaldehyde for 15 min and stained with crystal violet and quantified under a light microscope (Leica).

### 2.7. MTT Assay

The MTT assay was performed as previously described [28]. Briefly, osteoblasts were seeded into 96-well plates (3 × 10^3^/well) and then incubated with LPA (20 *μ*M) or LPA analogue (20 *μ*M) for indicated time. At the end of incubation, 20 *μ*L of MTT (5 mg/mL) was added and incubated for 4 h. The supernatant was then removed, and 150 *μ*L dimethyl sulfoxide (DMSO) was added to each well. The absorbance of each well was determined at 490 nm and data were presented as a percentage of the control.

### 2.8. RNA Isolation and Real-Time Quantitative Polymerase Chain Reaction (PCR) Analysis

RNA isolation and real-time PCR were performed as previously described [29]. Briefly, total RNA of osteoblasts in each group was isolated using the TRIzol regent (Invitrogen) according to the manufacturer recommended protocol. Then cDNA was synthesized from 1 microgram of total RNA using the Revert Aid Fist strand cDNA Synthesis kit (# K1622, Thermo Scientific). The obtained cDNA was then amplified via real-time PCR with an ABI 7900 HT Real-Time PCR System (Applied Biosystems) and SYBR® GREEN Real-time PCR Master Mix (TOYOBO, Japan). Glyceraldehydes-3-phosphate dehydrogenase (GAPDH) was used as normalized gene. Results are represented as a fold change of the comparative expression level. The sequences of the forward and reverse primers were as follows: 5′-ATCACGAAGAAGCCATCGAG-3′ and 5′-TGTTCCCGAAAAATCTGGAG-3′ for BMP2 gene; 5′-TGCCCAGGCGTATTTCAG-3′ and 5′-TGCCTGGCTCTTCTTACTGAG-3′ for RUNX2 gene; 5′-CTCATGGAGGCCTTTGTCTT-3′ and 5′-CTCATGATGTCCGTGGTCAA-3′ for ALP gene; 5′-AGCAGCTTGGCCCAGACCTA-3′ and 5′-TAGCGCCGGAGTCTGTTCACTAC-3′ for OCN gene; and 5′-ACCACAGTCCATGCCATCAC-3′ and 5′-TCCACCACCCTGTTGCTGTA-3′ for internal control GAPDH gene. The primers used in our present was synthesized by Sangong Biotech Co., Ltd (Shanghai, China), expert in primer synthesis. And these primers have been previously validated by Sangong Biotech before delivery.

### 2.9. Protein Extraction and Western Blot Analysis

After treatment with LPA (20 *μ*M) or LPA analogue (20 *μ*M) for 72 h, osteoblasts were washed with PBS buffer three times and then treated with RIPA Lysis Buffer. Cell lysates were clarified by centrifugation and protein concentration in the supernatants was measured by the BCA Protein assay (Thermo Scientific). After that, 20 *μ*g total proteins were separated by 10% SDS-polyacrylamide gels. Proteins were then transferred onto PVDF membranes. The immunoblots were blocked with 5% (w/v) nonfat milk and incubated with primary antibodies at dilutions recommended by the suppliers in TBST buffer overnight at 4°C. Protein expression was detected by using an enhanced chemiluminescence reagent (Thermo Scientific). *β*-Actin was used as internal control protein.

### 2.10. Blood Analysis

Age- and weight-matched healthy female C57BL/6 mice were intravenously injected with PBS (200 *μ*L, n=6), LPA (20 *μ*M, 200 *μ*L, n=6), or LPA analogue (20 *μ*M, 200 *μ*L, n=6) via the tail vein. The body weights of the mice were measured every week. Ten weeks later, the mice were euthanized, and approximately 500 *μ*L blood samples were collected from each mouse. The blood samples were used for blood chemistry tests and whole blood cell analyses. The organs were harvested and fixed in 4% buffered paraformaldehyde for 24 h. The fixed organs were used for histological analysis.

### 2.11. Biomechanical Characterization Testing

Compression testing and three-point loading were preformed according to our previous study [30].

### 2.12. Mineralization Assay

To investigate the osteoblastic differentiation effect of LPA and LPA analogue, the fourth-generation femur osteoblasts were cultured in *α*-MEM medium containing 10% FBS, 100 *μ*g/mL penicillin/streptomycin, 50 *μ*g/mL L-ascorbic acid (Sigma-Aldrich), 10^−7^ dexamethasone (Sigma-Aldrich), and 10 mM *β*-glycerophosphate (Sigma-Aldrich) for 28 days. Cells in experimental group were cultured in osteogenic inducing conditional media supplemented with LPA (20 *μ*M) or LPA analogue (20 *μ*M). Mineralized nodules were visualized by Alizarin Red S staining and quantitated by bromohexadecyl pyridine (Aladdin). To quantify the mineralized nodule formation, the Alizarin Red S was extracted by with 10% cetylpyridinium chloride and the absorbance in each group was determined at 520 nm and data were presented as a percentage of the control.

### 2.13. Animals and Surgery Procedure

Animals were obtained from the Experimental Animal Center of Wuhan University. We declare that all animal procedures were approved by the Animal Care and Use Committee of the Hospital of Stomatology, Wuhan University. Forty-five mature female C57BL/6 mice (10 weeks old and mean body weight 22.7 g) were used. All the animals underwent bilateral osteotomy surgery according to the surgery procedure described previously [30]. Briefly, when the femurs were exposed, plates were placed on the femurs and fixed by 4 screws. Then a segmental osseous defect of 2.0 mm was created and the width was notarized by inserting the 2.0 mm scale plate into the osteotomy gaps. After flushing with saline solution. The left femur osteotomy gap was filled with our self-made porous scaffolds of degradable chitosan/beta-tricalcium phosphate [31] containing LPA (200 *μ*M, 20 *μ*L) or LPA analogue (200 *μ*M, 20 *μ*L). The surgery area was closed in layers and the animals take food and water freely in the experimental period. Two, five, and ten weeks after surgery, five mice were sacrificed, respectively, by an overdose of anesthetics and femurs were harvested and prepared for analysis.

### 2.14. Micro-Computed Tomography (Micro-CT) Assay

All the femurs were fixed in 4% paraformaldehyde solution for 24 h at 4°C. A Micro-CT imaging 50 system (Scanco Medical, Bassersdorf, Switzerland) was used to evaluate bone formation within the femurs osteotomy gap. The scanning parameters were performed (70 kV, 114 *μ*A, and 50 *μ*m per slice).

### 2.15. Histological Analysis

Femurs samples were decalcified using 10% ethylenediaminetetraacetic acid (EDTA) solution (PH: 7.2) for 2 weeks on a shaker at room temperature. The EDTA solution was changed twice per week. Femurs samples and other organs were dehydrated in a series of graded concentrations of ethanol from 70% to 100%. Then, 6 *μ*m thick longitudinal serial sections were cut and stained with hematoxylin and eosin (H&E).

### 2.16. Statistical Analysis

Data were analyzed with one-way ANOVA using GraphPad Prism 5.0. All data were expressed in mean ± standard error (SEM). The level of significance was p<0.05 unless otherwise stated.

## 3. Results

### 3.1. LPA and LPA Analogue Promoted Osteoblasts Growth and Migration

**Figure 1:**
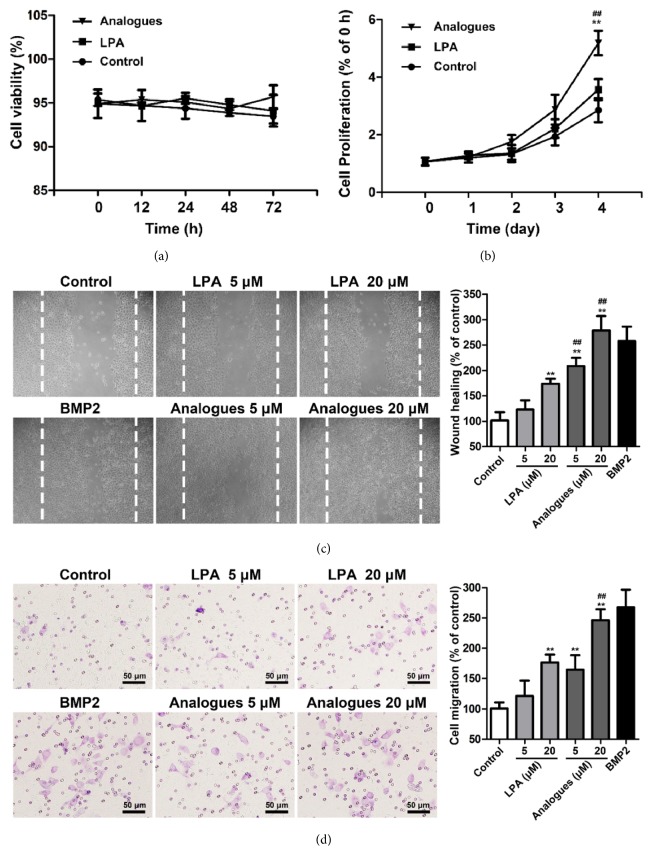
The effect of LPA and LPA analogue on viability, proliferation, and migration of osteoblast. (a) Viability of osteoblasts after being exposed to LPA (20 *μ*M) or LPA analogue (20 *μ*M) for 0-72 h. (b) Proliferation of osteoblasts after incubation with LPA (20 *μ*M) or LPA analogue (20 *μ*M) for 0-4 days. (c) The migration of osteoblasts was determined by wound-healing assay. The dashed lines were the borders of the original scratch. (d) The migration of osteoblasts was evaluated by transwell assay. *∗∗*p < 0.01 compared with control group. ^##^p < 0.01 compared with LPA group.

To evaluate the effects of LPA and LPA analogue on osteoblasts, we employed primary osteoblasts for in vitro studies. As shown in Figure 1(a), LPA and LPA analogue have no cytotoxicity to osteoblasts when incubated with cells even for 72 h. In addition, no morphological changes have been observed during the incubation periods (Supplementary Materials, Figure S1). Results of MTT assay demonstrated that the growth activity of osteoblasts was not significantly changed by incubation with LPA for 4 days (Figure 1(b)), which were in line with our previous report [17]. However, compared with control and LPA group, the growth activity of osteoblasts was significantly enhanced by incubation with LPA analogue, suggesting the marked proliferation-promoting effect on osteoblasts. It is reported that the migration of osteoblasts precursor cells into the fracture gaps is an essential step during bone fracture healing process [4]. Our results of wound-healing assay and transwell migration assays showed that the mobility of osteoblasts was significantly increased by LPA and LPA analogue in a concentration-dependent manner (Figures 1(c) and 1(d)). Importantly, LPA analogue has greater influence on the migration of osteoblasts than that of LPA. BMP2, which could significantly promote the migration of osteoblasts [32], was used as a positive control.

### 3.2. LPA and LPA Analogue Promoted Osteogenic Differentiation and Mineral Nodule Formation In Vitro

**Figure 2:**
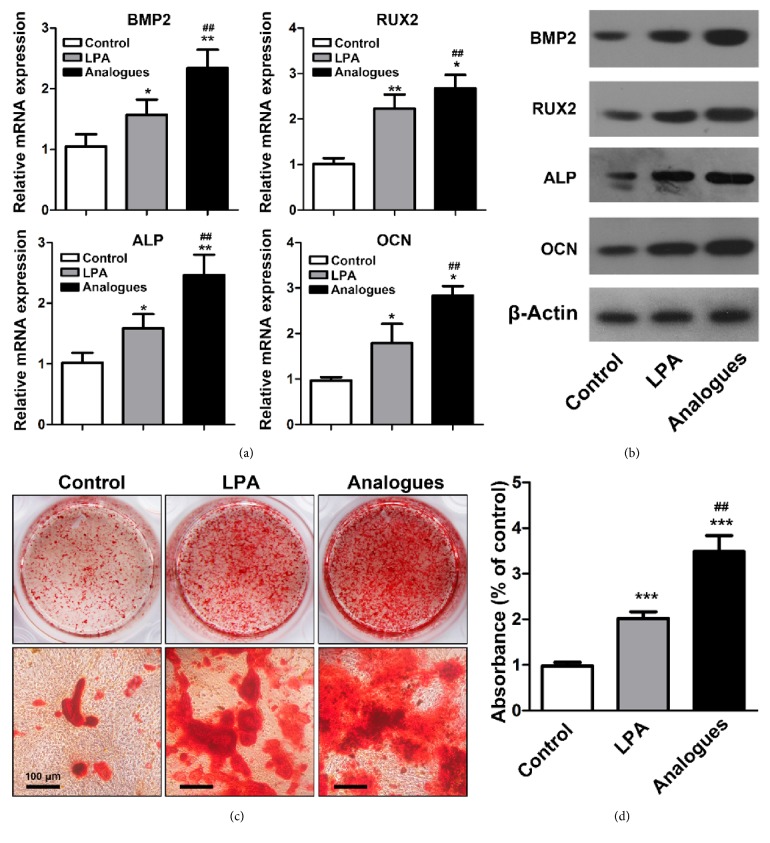
LPA and LPA analogue promoted osteogenic differentiation and mineral nodule formation in vitro. The expression level of osteogenic markers in osteoblasts treated by LPA or LPA analogue was determined by real-time PCR (a) and western blot (b). (c) The mineralized nodules were visualized by Alizarin Red staining. (d) Mineralized nodules were quantitated by cetylpyridinium chloride and the absorbance was measured at 562 nm. *∗*p < 0.05 compared with control group. *∗∗*p < 0.01 compared with control group. *∗∗∗*p < 0.001 compared with control group. ^##^p < 0.01 compared with LPA group.

To evaluate the effects of LPA and LPA analogue on differentiation of osteoblasts, the expression of ossification-related gene and protein expression were analyzed by incubating primary osteoblasts with osteogenic culture medium in the absence of LPA or LPA analogue for 4 and 14 days. Data of real-time PCR revealed that LPA and LPA analogue increased the mRNA expression levels of BMP2, ALP, and RUNX2 on day 4 and OCN expression on day 14 (Figure 2(a)). Those enhancements were confirmed by western blot assay (Figure 2(b)). It should be noted that the expression levels of mRNA and protein tested in cells treated with LPA analogue were significantly higher than LPA-treated cells. Then, we evaluated the effect of LPA and LPA analogue in mineralized matrix formation of osteoblasts in vitro. The mineralized nodules were visualized by Alizarin Red S staining after 28 days of treatments (Figure 2(c)). More red mineral nodules were observed in the LPA and LPA analogue group than the control group and the strongest mineral staining appeared in LPA analogue-treated group. Quantification of Alizarin Red S staining (Figure 2(d)) also verified that LPA analogue significantly enhanced the mineral deposition in osteoblasts compared with LPA and control group.

### 3.3. Biocompatibility of LPA and LAP Analogue In Vivo

**Figure 3:**
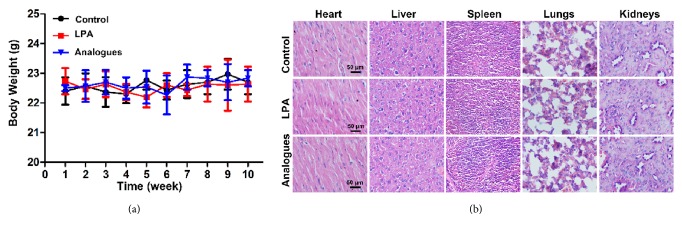
Biocompatibility of LPA and LPA analogue in vivo. (a) Weekly changes of body weight in testing animals during 10 weeks. (b) H&E stained images of major organs harvested from mice injected with PBS, LPA, and LPA analogue, respectively.

To evaluate the biosafety of LPA and LPA analogue in vivo, LPA analogue (20 *μ*M, 200 *μ*L) or LPA (20 *μ*M, 200 *μ*L) was injected into mice via tail vein. Body weight of mice from LPA and LPA analogue-treated groups did not differ significantly from that of control group (Figure 3(a)). Ten weeks after injection, mice were euthanized for blood biochemistry, hematology analyses, and histological examinations. Sex-, age-, and weight-matched healthy mice were used as controls. Intravenous injection of LPA analogue did not lead to reductions in hepatic or renal function, as demonstrated by the unaltered indicators for liver and kidney functions (Table 1). Furthermore, there was no statistically significant difference in indicators for blood cells, hemoglobin, and platelets determined by hematology analyses (Table 2). In addition, neither apparent organ injury nor inflammation alteration was observed in the heart, liver, spleen, lungs, and kidneys harvested from LPA or LPA analogue-treated mice when compared with the control group, indicating negligible histological toxicity of LPA and LPA analogue (Figure 3(b)).

### 3.4. LPA Analogue rather than LPA Promoted the Bone Formation In Vivo

**Figure 4:**
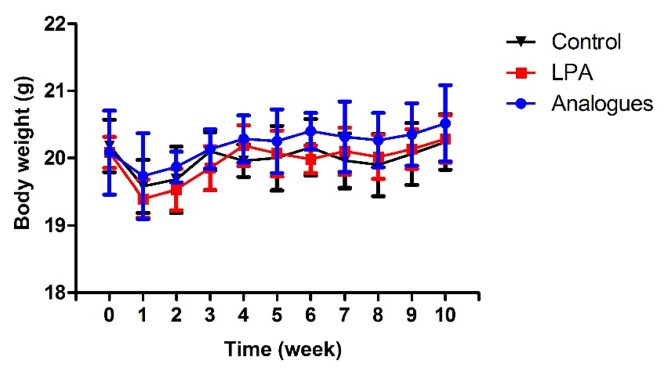
Weekly changes of body weight in testing animals after surgical operation.

With favorable biocompatibility and powerful enhancement in osteogenic differentiation, LPA and LPA analogue have shown great potential for bone healing in bone fracture in vivo. We then evaluate the bone formation in murine femoral defect model treated with LPA or LPA analogue. LPA was absorbed on porous scaffolds of degradable chitosan/beta-tricalcium phosphate [31]. Then, porous scaffolds containing LPA or LPA analogue were inserted into the osteotomy gap. There was no difference between control and LPA group in food intake after osteotomy. The body weight of animals in control decreased from weeks 1-2 but gradually increased from weeks 2-5. Similar trends was also observed in LPA-treated group (Figure 4).

**Figure 5:**
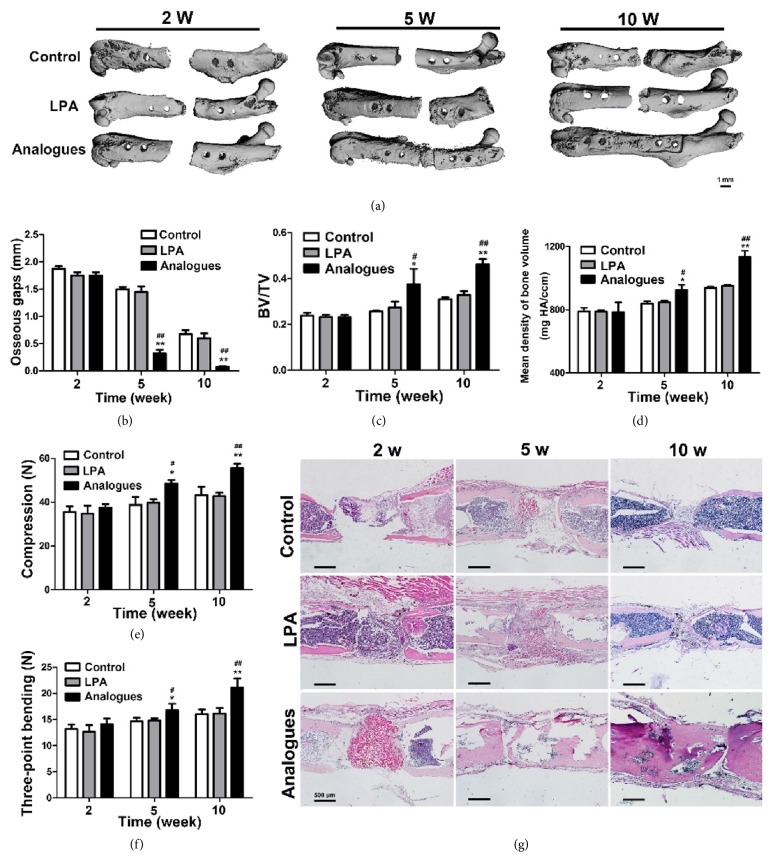
LPA analogue rather than LPA promoted the bone formation in vivo. (a) Micro-CT 3D reconstruction images of LPA and LPA analogue-treated group at different time points. (b) Changes of osseous gaps in testing animals at the end of 2, 5, and 10 weeks. (c) Results from Micro-CT of the ROI for bone volume over total volume (BV/TV) at different time points. (d) Mean density of bone volume of LPA and LPA analogue group at different time points. (e) The compressive strength of femur from LPA analogue group was greater than control and LPA group. (f) Three-point bending of femur from LPA analogue group was greater than control and LPA group. (g) H&E staining of LPA and LPA analogue group at 2, 5, and 10 weeks after operation (bar=500 *μ*m).

Micro-CT 3D reconstruction analyses (Figure 5(a)) showed that the two edges of the bone gap of control group and LPA group were sharp and parallel to each other at the end of 2 weeks. Although no significantly changes of osseous gaps were observed between all groups (Figure 5(b)), the gaps of LPA analogue group were blunter than other groups. At the end of 5 weeks, no union was observed in control and LPA group, except that the edges of both groups became narrower and obscure. Instead, in LPA analogue group, the bone gaps were filled with bone tissue and the osseous gaps were significantly reduced compared with control and LPA group. Union could be observed at the end of 10 weeks in all the specimens of LPA analogue group, while there were still obvious bone defects in control and LPA group. The osseous gaps further decreased and almost could not be observed in LPA analogue group. However, no obvious difference was observed between control and LPA group. The osteotomy gap and the adjacent cortical bone and bone marrow between the two inner screws were defined as the regions of interest (ROI) for Micro-CT analysis. The Micro-CT data showed that BV/TV (Figure 5(c)) and mean density of bone volume (Figure 5(d)) were not significantly altered by LPA treatment. LPA analogue enhanced the bone formation which was indicated by the increased values of BV/TV and mean density of bone volume. The compressive strength and three-point bending of femur from LPA analogue group were greater than control and LPA group. There was no significant statistical difference between control and LPA group (Figures 5(e) and 5(f)). Results of HE staining showed that the bone gaps were filled with soft tissues and porous scaffolds in control, LPA, or LPA analogue-treated group two weeks after operation. At the end of 5 weeks, the osteotomy gaps became narrower and filled with soft tissues in control and LPA group. In contrast, although the gaps still remained, LPA analogue group formed osteoid in defect area. At the end of 10 weeks, the defect remained unhealed in control and LPA group. However, the defect of LPA analogue-treated femur was sufficiently close to be bridged by newly formed bone (Figure 5(g)). Those results demonstrated that LPA analogue promoted the bone formation in vivo, while LPA has no significant effect on bone formation.

## 4. Discussion

Fracture healing is an important and complex physiological process and the most important cells in fracture healing are osteoblasts. LPA is enriched in blood [9, 33] and it was critical regulator of skeletal biology and exerts effects through specific receptors expressed on osteoblasts [34]. It was reported that when applied to cells or tissues LPA is readily degraded to monoacylglycerol by phosphatase due to its chemical lability. In order to identify the effect of LPA, phosphatase-resistant LPA analogue was developed in the past decades. In present study, LPA and LPA analogue were used to evaluate the effect of LPA on bone formation in vitro and in vivo.

Osteoblasts proliferation and migration are considered important in bone healing. Our previous study reported that LPA did not affect the proliferation (Figure 1(b)), which was in line with the results of our present research. However, we found that LPA analogue significantly increased the growth activity of osteoblasts. Compared with LPA, LPA analogue has stronger capability to induce the migration of osteoblasts, which was demonstrated by wound healing and transwell assay (Figures 1(c) and 1(d)). Osteogenic markers including BMP2, RUNX2, ALP, and OCN in osteoblasts were significantly upregulated by LPA and LPA analogue (Figures 2(a) and 2(b)), in accordance with increased mineralized nodules in the Alizarin Red assay (Figure 2(c)). It is evident that LPA analogues have greater ability to induce the expression of marker genes and formation of mineralized nodules. Since the half-time of LPA in the cell culture was about 1 h while the concentration of LPA analogue was unchanged during the incubation [23], the discrepant effect of LPA and LPA analogue on proliferation, migration, and differentiation of osteoblasts may be attributed to the distinct performance of stability.

The ideal drugs should have low or even no toxicity to ensure biocompatibility of drugs in biomedical applications. Therefore, before in vivo application, we evaluate the system toxicity of LPA and LPA analogue. We found that LPA and LPA analogue did not reduce the osteoblasts viability and body weight of LPA or LPA analogue injected mouse. Moreover, the blood analysis and histological analysis confirmed the healthy status of LPA and LPA analogue-treated mice, revealing the excellent biocompatibility of LPA and LPA analogue in vitro and in vivo.

Since LPA and LPA analogue stimulated in vitro osteogenesis, we assessed the effect of LPA and LPA analogue on in vivo bone regeneration. To our surprise, although it can promote the mineralized matrix formation in vitro, we did not observe significant influence of LPA on bone formation, proved by Micro-CT and histological analysis (Figures 5(a)–5(g)). However, the bone healing process was significantly accelerated by LAP analogue. The degradation of LPA may result in the conclusion that LPA could not promote bone formation in vivo. In addition, when LPA was used in vitro, the culture medium was replaced by fresh culture medium containing fresh LPA every other day and the fresh LPA may play an important role before it was degraded. However, no fresh LPA will substitute the degraded LPA supplied through surgical operation when used in femur bone defect. This would explain the osteogenic induction of LPA when used in vitro while no effect on bone formation exists in vivo.

## 5. Conclusions

In summary, our study demonstrated that although LPA promotes mineralized matrix formation in vitro, LPA was not effective in promoting bone formation. Compared with LPA, LPA analogue was a potent stimulating factor to induce bone formation in vivo due to its excellent stability.

## Figures and Tables

**Table 1:** Whole blood cell analysis of mice after treatment with LPA or analogues.

**Index**	**Control**	**LPA**	**Analogues**
**WBC (×10** ^**9**^ **/L)**	6.33 ± 1.57	6.33 ± 2.01	6.27 ± 1.81
**Lymph (×10** ^**9**^ **/L)**	1.85 ± 0.28	1.80 ± 0.22	1.87 ± 0.24
**Mon (×10** ^**9**^ **/L)**	0.35 ± 0.19	0.30 ± 0.14	0.30 ± 0.14
**Gran (×10** ^**9**^ **/L)**	3.26 ± 0.52	3.16 ± 1.01	2.90 ± 0.16
**Lymph (%)**	34.47 ± 8.91	38.20 ± 9.38	33.70 ± 2.21
**Mon (%)**	4.98 ± 0.84	4.73 ± 1.06	4.97 ± 0.19
**Gran (%)**	59.62 ± 7.60	56.73 ± 10.10	59.33 ± 3.45
**RBC (×10** ^**12**^ **/L)**	8.98 ± 0.52	9.04 ± 0.53	9.09 ± 0.30
**HGB (g/L)**	141.00 ± 5.81	137.00 ± 2.16	143.67 ± 9.53
**HCT (%)**	46.13 ± 3.23	41.73 ± 8.06	46.63 ± 4.69
**MCV (fL)**	48.27 ± 3.93	48.67 ± 0.45	46.50 ± 4.81
**MCH (pg)**	15.57 ± 2.25	15.77 ± 1.51	15.20 ± 0.78
**MCHC (g/L)**	316.30 ± 5.80	303.30 ± 21.10	314.33 ± 2.36
**RDW (%)**	15.87 ± 2.81	15.57 ± 2.25	15.17 ± 0.57
**PLT (×10** ^**9**^ **/L)**	809.67 ± 80.5	767.33 ± 51.65	836.67 ± 226.18
**MPV (fL)**	5.52 ± 0.82	5.97 ± 0.70	5.83 ± 0.21
**PDW**	17.53 ± 0.35	17.43 ± 0.33	17.50 ± 0.22
**PCT (%)**	0.38 ± 0.17	0.39 ± 0.11	0.35 ± 0.15

WBC: white blood cell; Lymph: lymphocyte; Mon: monocyte; Gran: granulocyte; RBC: red blood cell; HGB: hemoglobin; HCT: hematocrit; MCV: mean corpuscular volume; MCH: mean corpuscular hemoglobin; MCHC: mean corpuscular hemoglobin concentration; RDW: red blood cell distribution width; PLT: platelets; MPV: mean platelet volume; PDW: platelet distribution width; PCT: plateletcrit.

**Table 2:** The hepatic and renal functions of mice after treatment with LPA or analogues were determined by blood chemistry tests.

**Index**	**Control**	**LPA**	**Analogues**
**TP (g/L)**	35.97 ± 0.25	36.10 ± 1.17	35.73 ± 1.43
**ALB (g/L)**	27.00 ± 2.27	26.80 ± 0.96	26.77 ± 0.74
**GLOB (g/L)**	9.06 ± 1.55	9.01 ± 1.22	9.17 ± 0.86
**A/G**	3.09 ± 0.51	3.03 ± 0.38	2.95 ± 0.29
**ALT (U/L)**	55.00 ± 3.74	53.33 ± 3.57	51.67 ± 5.78
**ALP (U/L)**	5.07 ± 2.12	5.17 ± 0.17	5.05 ± 0.13
**AST (U/L)**	104.73 ± 6.70	100.43 ± 3.63	103.33 ± 9.57
**BUN (mmol/L)**	6.58 ± 0.05	6.50 ± 0.09	6.44 ± 0.12
**CRE (*μ*mol/L)**	39.13 ± 2.12	37.80 ± 1.61	37.77 ± 3.07

TP: total protein; ALB: albumin; GLOB: globulin; A/G: albumin/globulin ratio; ALT: alanine aminotransferase; AST: aspartate aminotransferase; ALP: alkaline phosphatase; BUN: blood urea nitrogen; CRE: creatinine.

## Data Availability

The data used to support the findings of this study are available from the corresponding author upon request.

## Supplementary Materials

Supplementary Materials Figure S1: morphological observation of osteoblasts after incubation with LPA and LPA analogues for 3 days.
