# Supplementary material for: Lysophosphatidic Acid Analogue rather than Lysophosphatidic Acid Promoted the Bone Formation In Vivo
Source: Biomed Res Int. 2018 May 29;2018:7537630. doi: 10.1155/2018/7537630 (PMC5996417; doi:10.1155/2018/7537630)
Supplement: Supplementary Materials — Figure S1: morphological observation of osteoblasts after incubation with LPA and LPA analogues for 3 days. [file 7537630.f1.pdf]

Supplementary Material

For

Lysophosphatidic acid analogues rather than lysophosphatidic acid promoted the bone formation in vivo

Zi-Li Yu[1, 2], DDS, MD, PhD, zili09@whu.edu.cn

Bin-Fang Jiao[3], Bachelor, 2012203040032@whu.edu.cn

Zu-Bing Li[1, 2,*] DDS, MD, PhD, lizubing@ whu.edu.cn .

1 The State Key Laboratory Breeding Base of Basic Science of Stomatology (Hubei-MOST) and Key Laboratory of Oral Biomedicine Ministry of Education, School and Hospital of Stomatology, Wuhan University. 237, Luoyu Road, Wuhan, PR China.

2 Department of Oral and Maxillofacial Surgery, School and Hospital of Stomatology, Wuhan University. 237, Luoyu Road, Wuhan, RP China.

3 Wuhan NO.1 Stomatological Hospital. 675, Jianshe Avenue, Wuhan, PR China.

***Corresponding author:** Zu-bing Li, Department of Oral and Maxillofacial Surgery, School and Hospital of Stomatology, Wuhan University, 237 Luoyu Road, Wuhan 430079, PR China. E-mail address: lizubing@ whu.edu.cn (Zu-Bing Li).

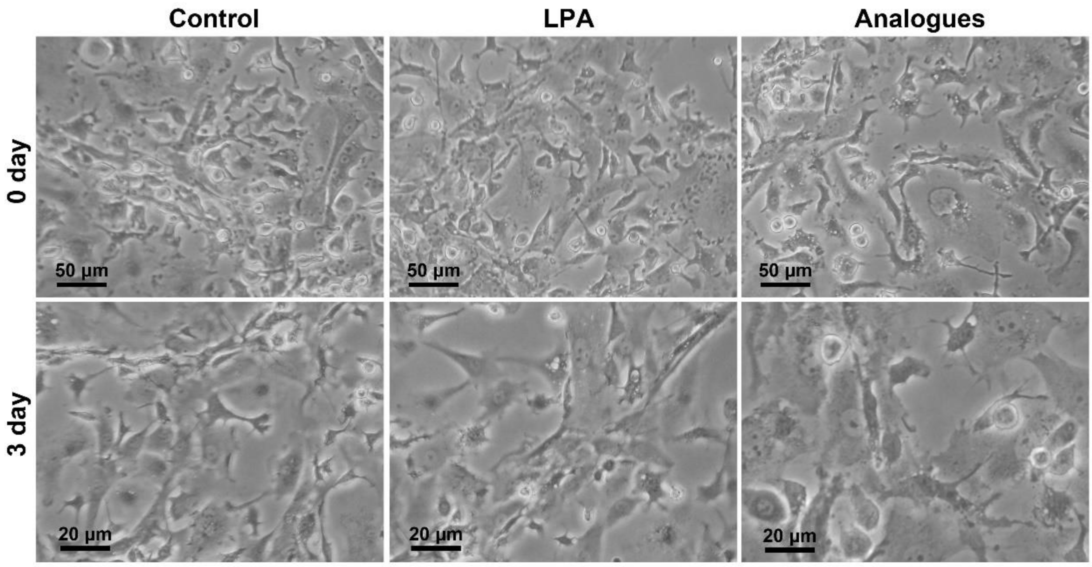

Figure S1 Morphological observation of osteoblasts after incubated with LPA and LPA analogues for 3 days.
